# Doxorubicin Plus Dacarbazine Versus Doxorubicin Plus Ifosfamide in Combination With Regional Hyperthermia in Patients With Advanced Leiomyosarcoma: A Propensity Score‐Matched Analysis

**DOI:** 10.1002/cam4.70655

**Published:** 2025-02-25

**Authors:** Luc M. Berclaz, Vindi Jurinovic, Anton Burkhard‐Meier, Sultan Abdel‐Rahman, Markus Albertsmeier, Alexander Klein, Hans Roland Dürr, Nina‐Sophie Schmidt‐Hegemann, Thomas Knösel, Wolfgang G. Kunz, Emanuel Stutz, Michael von Bergwelt‐Baildon, Dorit Di Gioia, Lars H. Lindner

**Affiliations:** ^1^ Department of Internal Medicine III, University Hospital LMU Munich Munich Germany; ^2^ German Cancer Consortium (DKTK) Partner Site Munich Munich Germany; ^3^ Institute for Medical Information Processing, Biometry, and Epidemiology, University Hospital LMU Munich Munich Germany; ^4^ Department of General, Visceral and Transplantation Surgery, University Hospital LMU Munich Munich Germany; ^5^ Orthopaedic Oncology, Department of Orthopaedics and Trauma Surgery, University Hospital LMU Munich Munich Germany; ^6^ Department of Radiation Oncology, University Hospital LMU Munich Munich Germany; ^7^ Institute of Pathology LMU Munich Munich Germany; ^8^ Department of Radiology, University Hospital LMU Munich Munich Germany; ^9^ Department of Radiation Oncology, Inselspital Bern University Hospital, University of Bern Bern Switzerland

**Keywords:** chemotherapy, leiomyosarcoma, regional hyperthermia, soft tissue sarcoma

## Abstract

**Background:**

Dacarbazine is currently considered the better combination partner for doxorubicin compared to ifosfamide for the treatment of leiomyosarcoma (LMS). Regional hyperthermia (RHT) combined with neoadjuvant chemotherapy has been shown to improve survival in patients with locally advanced high‐risk STS. We sought to evaluate the role of doxorubicin and dacarbazine (AD) versus doxorubicin and ifosfamide (AI) in combination with RHT in patients with LMS.

**Methods:**

Patients with locally advanced high‐grade LMS, including limited metastases, eligible for RHT and first‐line treatment with either AI + RHT or AD + RHT between 2014 and 2022 were retrospectively evaluated. Endpoints were progression‐free survival (PFS) and overall survival (OS). Patients were matched using propensity scores, which were estimated with a logistic regression model accounting for tumor site, presence of metastasis, surgery, and radiotherapy.

**Results:**

A total of 105 patients were included in this study, of which 101 were included in the propensity score‐matched cohort. In the matched cohort, treatment with AD + RHT was associated with a significantly improved PFS (HR 0.32, 95% CI 0.13–0.74, *p* = 0.0081). Multivariable analysis revealed several significant predictors of PFS, including treatment with AD + RHT (HR 0.42, 95% CI 0.19–0.92, *p* = 0.031).

**Conclusion:**

Treatment with AD + RHT showed improved PFS and better treatment tolerability compared to AI + RHT. Our results support the use of AD instead of AI for the treatment of patients with LMS in combination with RHT.

## 1 Introduction

Soft tissue sarcomas (STS) are rare tumors with multiple distinct histopathological subtypes. They account for approximately 1% of adult malignancies [1]. Leiomyosarcoma (LMS) is among the most common histological subtypes, accounting for 10%–20% of all STS. In patients with LMS and high‐risk features (G2/G3, ≥ 5 cm, deep to the fascia), prognosis is poor [2, 3]. Due to their aggressive behavior, high‐grade uterine and extra‐uterine LMS show a high risk of recurrence of 50%–70% or approximately 40% despite complete resection [2, 4, 5]. Therefore, in locally advanced LMS, chemotherapy in addition to a radical surgical approach may be proposed for fit patients affected by disease at high risk of death, often described as a 10‐year overall survival (OS) of < 60% [6, 7]. For patients with advanced and metastatic LMS, chemotherapy is based on anthracyclines as a monotherapy or in combination with dacarbazine (AD), or in combination with ifosfamide (AI), with current data suggesting a limited effect of AI [8, 9, 10, 11, 12, 13]. More recently, the combination of doxorubicin and trabectedin followed by trabectedin maintenance demonstrated favorable activity as first‐line therapy for metastatic and unresectable LMS [14, 15]. In contrast, the first‐line combination of gemcitabine and docetaxel failed to demonstrate superiority compared to anthracycline‐based therapies [16, 17, 18, 19].

As described in the ESMO‐EURACAN‐GENTURIS guidelines on STS, the addition of regional hyperthermia (RHT) to neoadjuvant chemotherapy is a feasible option [6]. This is mostly due to a large multicenter EORTC Phase III study that demonstrated a 10‐year OS benefit of 9.9% with chemotherapy and RHT compared to chemotherapy alone [20, 21]. Synergistic effects with RHT are related to improved drug delivery into the tumor, increased drug cytotoxicity, direct thermal toxicity, and potential tumoricidal immune responses by increasing immune infiltrates and priming the tumor microenvironment [22, 23, 24]. The synergistic effect of RHT in combination with ifosfamide is well established, showing a thermal enhancement ratio from 1.52 to 3.6 [22, 25, 26, 27]. In contrast, there is only limited data on the effect of RHT on the efficacy of dacarbazine [28, 29]. In our institution, patients with locally advanced high‐grade STS, including limited metastases, have been routinely treated with pre‐ or postoperative/additive chemotherapy with AI and RHT. In the year 2020, our standard protocol for LMS was changed to AD + RHT due to the promising results in the multicenter analysis conducted by D'Ambrosio et al., which showed a favorable activity of AD in terms of overall response rate (ORR) and progression‐free survival (PFS) compared to AI and doxorubicin alone [8]. In this study, we sought to compare both protocols in a large and well‐characterized cohort.

## 2 Materials and Methods

### 2.1 Patient Selection

An exploratory retrospective cohort study design was chosen to address the research question. Eligible patients were ≥ 18 years of age and had pathologically confirmed LMS with high‐risk features (G2/G3, deep to the fascia). Clinical, pathological, and outcomes data were extracted from our clinical sarcoma database. Patients received a multimodal first‐line treatment including up to eight cycles of doxorubicin‐based chemotherapy in combination with either dacarbazine or ifosfamide between 2014 and 2022. Patients under 60 years of age received 60 mg/m^2^ of doxorubicin per cycle and 9 g/m^2^ of ifosfamide (reduced to 6 g/m^2^ AI for cycles 5–8) or 1200 mg/m^2^ of dacarbazine. The standard dose for patients older than 60 years was 60 mg/m^2^ of doxorubicin combined with either 900 mg/m^2^ of dacarbazine or 6 g/m^2^ of ifosfamide per cycle. The doxorubicin dose of 60 mg/m^2^ was chosen to increase the number of chemotherapy cycles without reaching the maximal cumulative anthracycline dose while maximizing the local effect of RHT [21]. All patients were treated with chemotherapy in combination with RHT. RHT aiming for tumor temperatures elevating to 42°C–43°C for 60 min was given twice per AI or AD cycle. Quality and safety of hyperthermia were ensured by the European Society for Hyperthermic Oncology (ESHO) guidelines [30]. The BSD‐2000 hyperthermia system (PYREXAR Medical, Salt Lake City, UT, USA) was used. In patients with locally advanced disease, surgery was generally performed after four cycles of chemotherapy and RHT following discussion in our interdisciplinary tumor board. Patients with initial tumor resection received the complete treatment in an adjuvant (R0) or additive (R1/R2) approach. Radiotherapy was used in a pre‐ or postoperative setting in patients with extremity sarcomas or in selected nonextremity cases to enhance local tumor control. In addition to chemotherapy and RHT, patients with evidence of limited metastases eligible for RHT were discussed in our interdisciplinary tumor board to evaluate potential local ablative therapies including radiotherapy and surgery. Patients who received both protocols (e.g., switch from AI to AD due to impaired renal function) had severe or uncontrolled concomitant disease or patients who had previously received chemotherapy were excluded from the analysis.

### 2.2 Outcomes

The primary objective of this study was to explore the impact of AD and AI in combination with RHT as a first‐line treatment for locally advanced or metastatic LMS. Endpoints of this study included PFS and OS. The PFS duration was estimated by the time from the start of chemotherapy and RHT or surgery to first local or distant progression. OS was estimated by the time from the start of chemotherapy to death. In patients receiving preoperative chemotherapy, radiologic tumor response was assessed according to the RECIST 1.1 criteria after two cycles of chemotherapy [31]. RECIST evaluations were obtained by measuring the tumor's largest diameter, and measures were performed on the most convenient CT or MR sequence available. Outcomes were compared with radiologic response to chemotherapy. Because of the absence of randomization, matching of patients was performed by using a propensity score.

### 2.3 Statistical Analysis

Continuous variables were compared using the Mann–Whitney *U* test and categorical variables with the Fisher's exact test. Survival analyses were performed with Cox proportional hazards regression. The propensity score was estimated with logistic regression, and the data were matched with the optimal full matching method, in which all subjects received at least one match [32]. All *p*‐values ≤ 0.05 were considered significant.

All statistical analyses were done using the statistical software R (version 4.4.1). The R package *MatchIt* (version 4.5.5) was used for propensity score matching and the package *adjustedCurves* (version 0.11.2) for plotting the adjusted Kaplan–Meier curves.

## 3 Results

### 3.1 Patient Cohort

**FIGURE 1:**
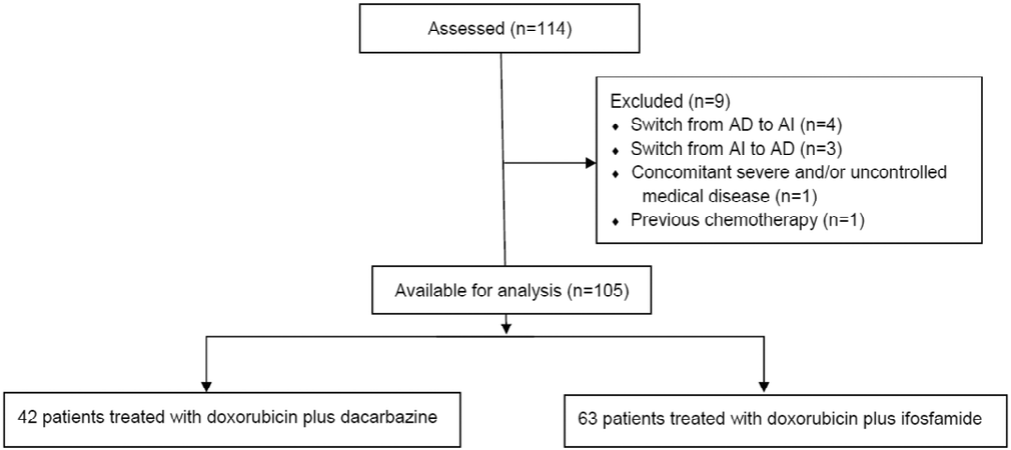
Flowchart of evaluated patients.

One hundred and five patients treated between 2014 and 2022 were analyzed (Figure 1). The patient and tumor characteristics of the study cohort are summarized in Table 1. Sixty‐three patients were treated with AI + RHT, whereas 42 patients received AD + RHT. The rate of metastasis at the beginning of chemotherapy was higher in the AD + RHT group (50% vs. 19%, *p* = 0.0012). LMS of the extremity was more common in the AI + RHT group (21% vs. 2%, *p* = 0.0020). Furthermore, surgery was more often performed in the AI + RHT group (97% vs. 81%, *p* = 0.013). More patients received pre‐ or postoperative radiotherapy in the AI group due to the higher rate of extremity LMS (33% vs. 9%, *p* = 0.0044).

**TABLE 1:** Patient characteristics.

	Value		AI	AD	*p*‐value
Age		Median (range)	57 (34–77)	58 (25–83)	0.39
Sex	Female	*n* (%)	45 (71)	35 (83)	0.24
	Male		18 (29)	7 (17)	
Recurrent tumor	Yes	*n* (%)	12 (19)	12 (29)	0.34
	No		51 (81)	30 (71)	
Largest diameter		Median (range)	10 (3–29)	10 (4–24)	0.79
Histology	Uterine LMS	*n* (%)	24 (38)	13 (31)	0.53
	Non‐uterine LMS		39 (62)	29 (69)	
Metastasis at beginning of chemotherapy	Yes	*n* (%)	12 (19)	21 (50)	0.0012
	No		51 (81)	21 (50)	
Grade	3	*n* (%)	31 (49)	22 (52)	0.84
	2		32 (51)	20 (48)	
Site of primary tumor	Extremity	*n* (%)	13 (21)	1 (2)	0.0085
	Intraabdominal		48 (76)	41 (98)	
	Trunk		1 (2)	0 (0)	
	Head and neck		1 (2)	0 (0)	
Site of metastases at beginning of chemotherapy	Liver	*n* (%)	2 (17)	7 (33)	0.21
	Pulmonary		8 (67)	6 (29)	
	Liver + pulmonary		0 (0)	2 (10)	
	Other		2 (17)	6 (29)	
Surgery	Yes	*n* (%)	61 (97)	34 (81)	0.013
	No		2 (3)	8 (19)	
Extent of surgery	R0	*n* (%)	40 (66)	19 (58)	0.66
	R1		11 (18)	9 (27)	
	R2		1 (2)	1 (3)	
	RX		9 (15x)	4 (12)	
Radiotherapy	Yes	*n* (%)	21 (33)	4 (9)	0.0044
	No		42 (67)	38 (91)	
Radiologic response to preoperative CT	PR	*n* (%)	8 (29)	5 (19)	0.52
	SD		16 (57)	13 (50)	
	PD		4 (14)	8 (31)	

### 3.2 Chemotherapy Data

The median number of chemotherapy cycles was similar in both treatment arms (median 8 cycles, range 1–8, *p* = 0.17) (Table 2). The number of dose reductions, mostly due to hematological toxicity, was significantly higher in the AI arm (33% vs. 10%, *p* = 0.0051). The rate of regular treatment completion after 8 cycles of chemotherapy and RHT was similar in both subgroups (60% vs. 51%, *p* = 0.078). The rate of treatment interruption due to disease progression was higher in patients with metastatic disease at the beginning of treatment (39% vs. 11%, *p* = 0.0014). Three patients in the AD arm began their treatment with chemotherapy only and therefore received a higher doxorubicin starting dose (75 mg/m^2^). The dose was reduced after the addition of RHT in the following cycles.

**TABLE 2:** Chemotherapy data.

	Value		AI	AD	*p*‐value
Chemotherapy cycles		Median (range)	8 (1–8)	7.5 (2–8)	0.17
Distribution of chemotherapy cycles in patients without surgery	1–4	*n* (%)	1 (50)	6 (75)	> 0.999
	5–7		0	0	
	8		1 (50)	2 (25)	
Preoperative chemotherapy	Yes	*n* (%)	28 (44)	26 (62)	0.11
	No		35 (56)	16 (38)	
Number of preoperative cycles		Median (range)	4 (1–8)	4 (2–8)	0.92
Distribution of preoperative chemotherapy cycles	1–3	*n* (%)	5 (19)	5 (28)	0.72
	4–8		21 (81)	13 (72)	
Postoperative chemotherapy	Yes	*n* (%)	50 (79)	27 (64)	0.12
	No		13 (21)	15 (36)	
Number of postoperative cycles		Median (range)	7.5 (2–8)	4 (1–8)	0.095
Distribution of postoperative cycles in patients with primary surgery	1–4	*n* (%)	5 (14)	3 (21)	0.72
	5–7		6 (17)	3 (21)	
	8		25 (69)	8 (57)	
Distribution of postoperative cycles in patients with surgery after chemotherapy	1–3	*n* (%)	5 (33)	5 (42)	0.71
	4–6		10 (67)	7 (58)	
Doxorubicin starting dose	60	*n* (%)	63 (100)	39 (93)	0.061
	75		0 (0)	3 (7)	
Ifosfamide starting dose (g/m^2^)	6	*n* (%)	34 (54)		
	9		29 (46)		
DTIC starting dose (g/m^2^)	900	*n* (%)		17 (40)	
	1200			25 (60)	
Dose reduction	Yes	*n* (%)	21 (33)	4 (10)	0.0051
	No		42 (67)	38 (90)	
Reason for dose reduction	Hematological toxicity	*n* (%)	20 (32)	3 (7)	0.0038
	Other toxicity		1 (2)	1 (2)	
Reason for interruption	Treatment completed	*n* (%)	38 (60)	22 (51)	0.078
	Disease progression		9 (14)	12 (29)	
	Toxicity		9 (14)	4 (10)	
	Physician's choice		5 (8)	0 (0)	
	Patient refusal		2 (4)	4 (10)	

### 3.3 Patient Outcomes in the Unmatched Population

**FIGURE 2:**
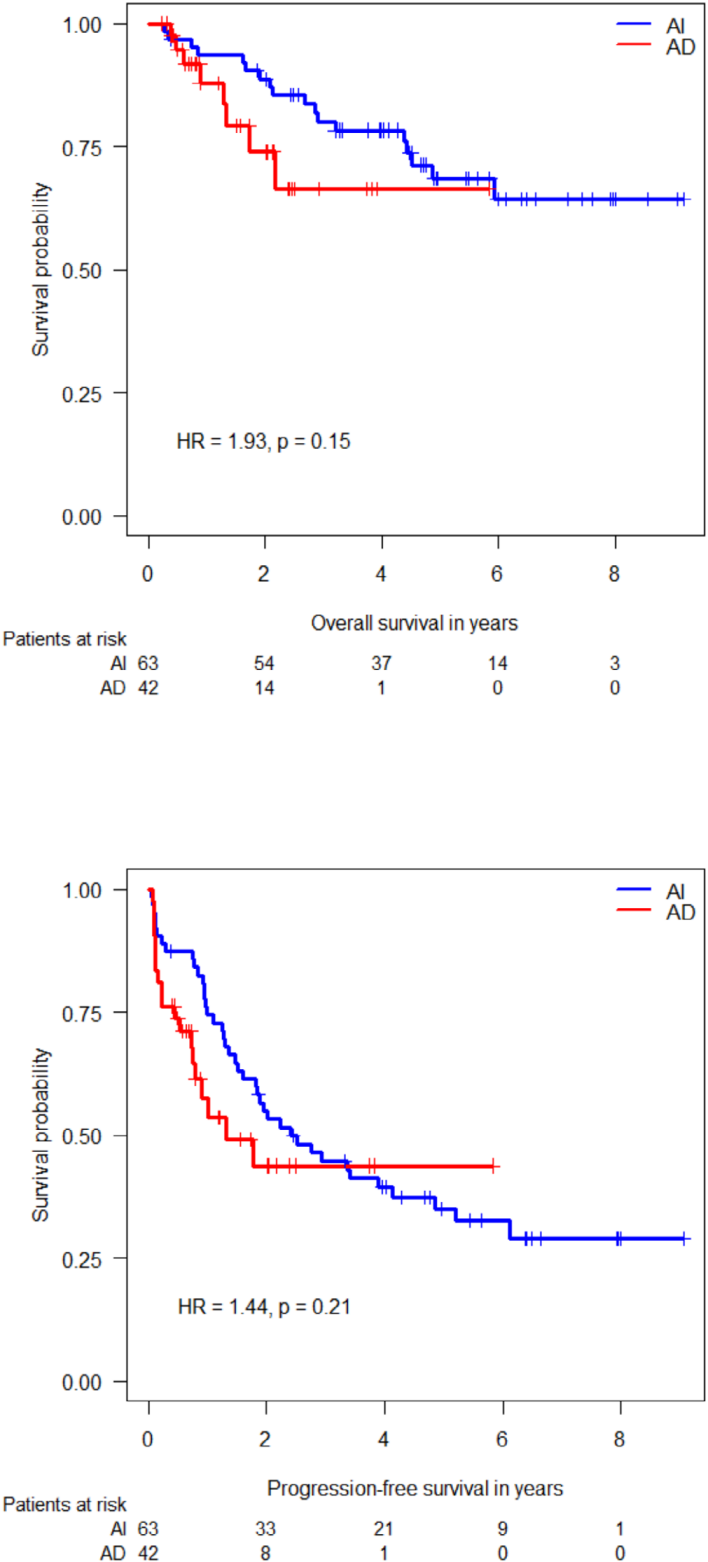
Overall survival (OS) and progression‐free survival (PFS) in the unmatched population.

For the 105 patients included in this study, the median follow‐up (FU) was 3.97 years. The overall 2‐year OS and 2‐year PFS were 84.5% (95% CI 77.2%–92.3%) and 50.3% (95% CI 41.0%–61.7%), respectively. The median FU was 1.57 years in the AD arm and 5.43 years in the AI arm. The 2‐year OS in the AI + RHT group was 88.7% (95% CI 81.2%–97.6%) versus 73.9% (95% CI 58.3%–93.6%) in the AD + RHT group, respectively (*p* = 0.21). The 2‐year PFS was 54.9% (95% CI 43.8%–68.8%) in the AI + RHT group versus 43.7% (95% CI 28.5%–67.0%) in the AD + RHT group (*p* = 0.115) (Figure 2). A trend toward poorer OS was visible in the AD + RHT group in patients receiving 1–4 cycles of chemotherapy (OS: HR 2.77, *p* = 0.073, PFS: HR 1.76, *p* = 0.18).

### 3.4 Patient Outcomes in the Matched Population

**FIGURE 3:**
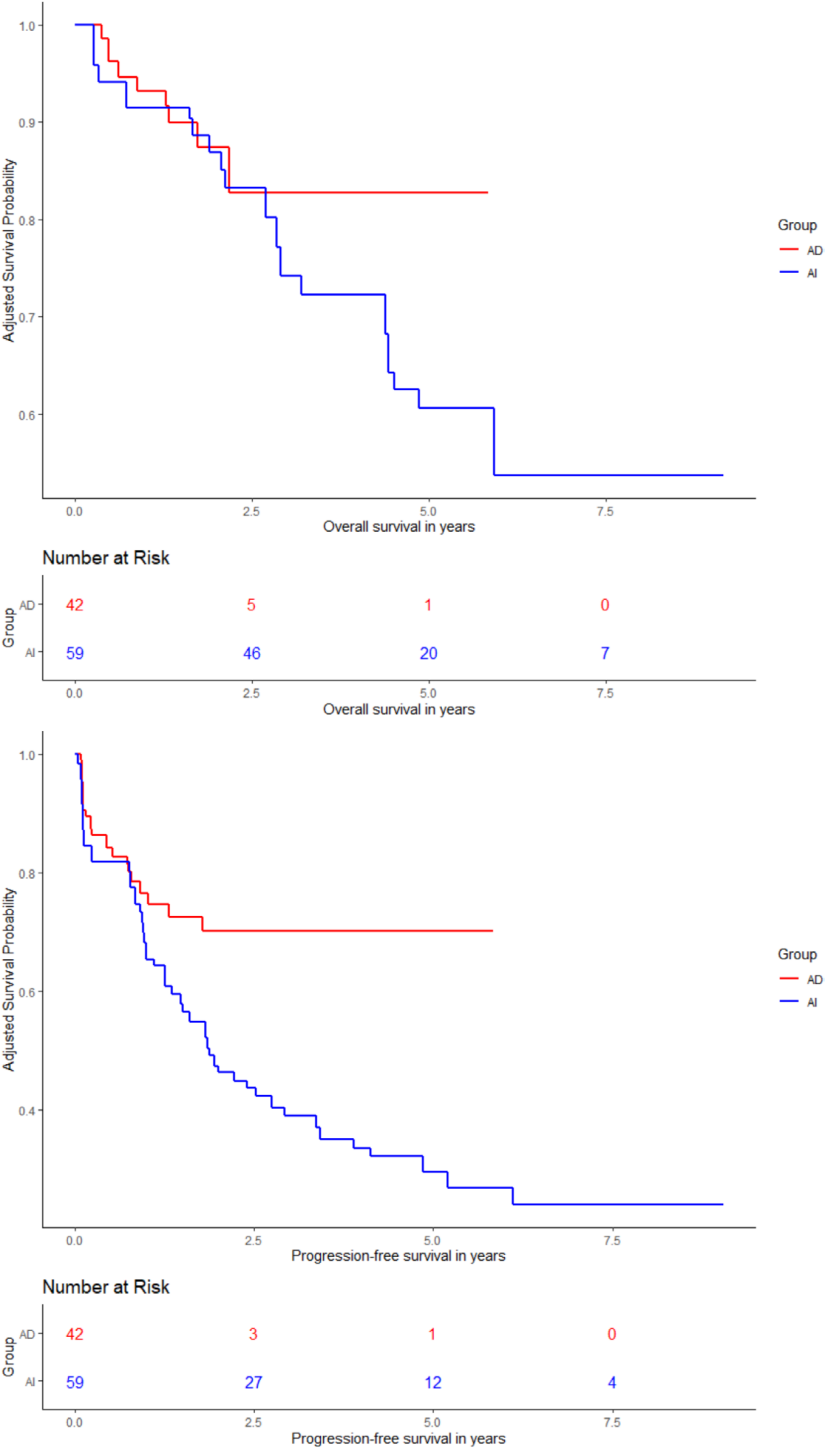
Overall survival (OS) and progression‐free survival (PFS) in the propensity score‐matched population.

Patients were matched according to their propensity scores, which were estimated with a logistic regression model accounting for significantly different baseline characteristics in both subgroups (tumor site, surgical resection, radiotherapy, and metastasis at the beginning of chemotherapy). Information on radiotherapy was missing for four patients; thus, the matched cohort consisted of 101 patients. The performance of the propensity score matching and the baseline characteristics of the matched cohorts are included in Table S1 and Figures S1–S5. Tumor site, metastasis at the beginning of chemotherapy, extent of surgery, and radiotherapy remained statistically significant predictors of PFS in the matched population (Table 3). The addition of radiotherapy was associated with both an improved local (HR 0.34, *p* = 0.0094) and distant PFS (HR 0.48, *p* = 0.016). Treatment with AD + RHT was significantly associated with a better PFS (HR 0.32, 95% CI 0.13–0.74, *p* = 0.0081). This effect was not maintained for OS (HR 0.43, 95% CI 0.11–1.73, *p* = 0.23) (Figure 3). In an exploratory subgroup analysis within the matched cohort, treatment with AD + RHT was not associated with better PFS/OS after separating the patients according to localized and metastatic disease (Tables S2–S5).

**TABLE 3:** Significant predictors of progression‐free survival (PFS) in the propensity score‐matched population.

Factor	Strata	Significance	Hazard ratio
Treatment regimen	AD vs. AI	0.0081	0.32 (0.13–0.74)
Tumor site	Extremity vs. non‐extremity	0.0024	0.45 (0.27–0.76)
Metastasis at the beginning of treatment	Yes vs. no	2.5∙10^−5^	4.35 (2.17–8.33)
Surgical resection	Yes vs. no	1.4∙10^−6^	0.18 (0.09–0.34)
Radiotherapy	Yes vs. no	0.020	0.60 (0.39–0.92)

### 3.5 Predictive Parameters

In the unmatched cohort, after adjustment for treatment groups, significant predictors of poor OS were the presence of metastasis at the beginning of chemotherapy (HR 3.45, 95% CI 1.50–7.95, *p* = 0.0036), larger tumor size (HR 1.08, 95% CI 1.02–1.15, *p* = 0.0081), progressive disease (PD) according to RECIST 1.1 (HR 13.0, 95% CI 1.56–108.51, *p* = 0.018), and incomplete resection (R1‐Rx vs. R0, HR 5.93, 95% CI 2.26–15.52, *p* = 0.00029). Regarding PFS, in addition to the presence of metastasis, PD according to RECIST, and incomplete resection, the presence of recurrent disease was predictive of a shorter PFS (HR 2.70, 95% CI 1.55–4.69, *p* = 0.00045). Radiotherapy was correlated with an improved PFS (HR 0.51, 95% CI 0.26–0.97, *p* = 0.041). The administration of 5–8 cycles of chemotherapy predicted a better overall and PFS compared to 1–4 cycles of chemotherapy (OS: HR 0.16, 95% CI 0.07–0.35, *p* < 0.0001; PFS: HR 0.25, 95% CI 0.14–0.42, *p* < 0.0001).

### 3.6 Multivariable Analysis

The results of multivariable analysis in the unmatched cohort are summarized in Table 4. Systemic therapy with AD + RHT, intermediate grade (G2) according to FNCLCC, and surgical resection were predictive of favorable PFS in the multivariable analysis. Poor PFS and a trend toward poor OS were visible in patients with the presence of metastasis at the beginning of systemic therapy.

**TABLE 4:** Multivariable analysis of progression‐free survival (PFS) and overall survival (OS).

Factor	Strata	PFS		OS	
		Significance	Hazard ratio	Significance	Hazard ratio
Treatment regimen	AD vs. AI	0.031	0.42 (0.19–0.92)	0.43	0.61 (0.18–2.08)
Age		0.14	1.02 (0.99–1.05)	0.27	1.03 (0.98–1.07)
Sex	Female vs. male	0.61	0.82 (0.39–1.75)	0.61	0.74 (0.23–2.34)
Histology	Uterine vs. non‐uterine	0.88	1.05 (0.53–2.07)	0.47	1.45 (0.53–3.96)
Grading	G2 vs. G3	0.031	0.50 (0.26–0.94)	0.33	0.64 (0.26–1.58)
Tumor site	Extremity vs. non‐extremity	0.17	0.53 (0.21–1.32)	0.64	0.71 (0.17–2.99)
Metastasis at beginning of treatment	Yes vs. no	0.00015	4.17 (1.99–8.73)	0.066	2.64 (0.94–7.44)
Surgery	Yes vs. no	0.042	0.36 (0.14–0.96)	0.088	0.30 (0.07–1.20)
Radiotherapy	Yes vs. no	0.23	0.61 (0.27–1.38)	0.35	0.52 (0.13–2.05)

## 4 Discussion

This is the first study to compare AD + RHT versus AI + RHT as a first‐line treatment in patients with locally advanced high‐grade LMS, including limited metastases. Our results demonstrate a PFS benefit and favorable toxicity profile in patients receiving AD + RHT. Historically, the addition of ifosfamide to anthracycline‐containing protocols did not have a significant impact on patient outcomes in LMS. Sleijfer et al. identified LMS patients as a group who benefited less from ifosfamide‐based therapy compared to doxorubicin alone [33]. In addition, Le Cesne et al. demonstrated an objective tumor regression in only 14% of LMS patients, which was exceptionally low compared to other histological subtypes [34]. D'Ambrosio et al. further questioned the effectiveness of ifosfamide and demonstrated the lowest response rate and median OS rates in patients with advanced and metastatic LMS receiving a combination of doxorubicin and ifosfamide [8]. Due to the retrospective nature of all mentioned studies and missing prospective trials, the final role of AI in LMS is still not fully understood. Conflicting results such as a trend toward improved PFS in the AI group compared to doxorubicin alone in the D'Ambrosio et al. study and missing differences in OS in the adjusted analyses make a correct interpretation difficult. In addition, patients only received a median number of 3 cycles of AI compared to 6 cycles of AD and tumor extent remained unbalanced in the AI arm after propensity score matching, which underlines basic differences in the patient cohorts. With many institutions involved in their multicenter study and therefore potentially center‐specific standards, there could be possible bias in the selection of patients for anthracycline monotherapy or the combination arms with ifosfamide or dacarbazine. The limited data on chemotherapy in LMS support the effort to conduct prospective trials of different anthracycline‐based chemotherapy regimens, such as the recent LMS04 trial, in patients with LMS [15].

An important difference in our study and why we hypothesized that AI + RHT might be of value in LMS is the synergistic role of RHT with alkylating agents such as ifosfamide. Ifosfamide is infused at a time point when the tumor has reached a temperature of 40°C–43.0°C, providing higher drug concentration due to increased perfusion together with increased cytotoxicity [27, 35]. On the other hand, there is only limited data on the cytotoxic effect of dacarbazine in concomitant application with RHT. Although preclinical data suggest a synergistic effect between dacarbazine and hyperthermia, the previous Phase‐III trial on the combination of chemotherapy and RHT only included patients treated with doxorubicin, ifosfamide, and etoposide [20, 22, 25, 26, 27, 28, 29]. The significant PFS benefit of AD + RHT in the multivariable analysis and the matched population and promising survival data in our patient cohort support current preclinical data and the potential regarding the combination of dacarbazine and RHT. A subsequent analysis with a larger and more homogenous patient cohort is necessary to confirm the value of AD + RHT in this patient subgroup.

The administration of 5–8 cycles of chemotherapy and RHT predicted a better OS and PFS compared to 1–4 cycles of therapy. The most common reasons for receiving fewer than 8 cycles of chemotherapy and RHT in our cohort were disease progression and treatment toxicity, which explains the better outcome in patients receiving a higher number of treatment cycles and an inevitable positive selection of patients in this subgroup. Our findings, however, pose a much‐debated question regarding the optimal number of neoadjuvant chemotherapy cycles in STS, including LMS. Gronchi et al. demonstrated a noninferiority of three preoperative cycles of AI chemotherapy compared to three preoperative and two postoperative AI cycles in localized high‐risk STS [36, 37]. D'Ambrosio et al. demonstrated a more favorable therapy adherence for AD versus AI, resulting in patients receiving a median number of 6 cycles of AD compared to a median number of 3 cycles of AI, which was associated with improved outcome and highlights our findings [8]. The optimal number of chemotherapy cycles with AD in patients with LMS should be evaluated in prospective trials.

Limitations of this study include the retrospective design and differences in baseline characteristics in both subgroups. FU in patients who received AD + RHT is limited, as our therapy protocol was switched from AI + RHT to AD + RHT in 2020, which results in a potential overestimation of survival outcomes in the AD arm. Data on OS are immature and will be presented with a longer FU in a subsequent analysis. In addition, as a significant number of patients with high‐risk LMS routinely underwent chemotherapy and RHT with curative intent and patients with diffuse metastatic disease more often received chemotherapy without RHT, treatment outcomes in this study are not easily comparable to previous literature on chemotherapy in more advanced and mostly metastatic LMS. The preliminary results of this study are hypothesis generating and should be interpreted with caution. On the basis of the results of this large single‐center analysis, we continue to apply AD + RHT in high‐risk LMS. In addition to the improved PFS in the propensity score‐matched population and the multivariable analysis, the lower rate of treatment toxicities is an important advantage of this regimen. A subsequent analysis with a longer FU, higher case numbers, and a more homogeneous cohort regarding metastasized vs. non‐metastasized patients and site of primary tumor should be conducted in the future.

## 5 Conclusion

In conclusion, the results of this study raise unanswered questions about the optimal systemic treatment of patients with advanced LMS and the potential effect of RHT in increasing the effectiveness of chemotherapy with AD. The combination of AD + RHT is well tolerated and potentially superior to AI + RHT, with promising results in the matched patient cohort and the multivariable analysis. A secondary analysis should be conducted in the future due to the limited FU in the patient group receiving AD + RHT. The nature of our results supports the effort to conduct prospective trials of different anthracycline‐based chemotherapy regimens in patients with LMS.

## Author Contributions

**Luc M. Berclaz:** conceptualization (equal), data curation (equal), formal analysis (equal), investigation (equal), methodology (equal), writing – original draft (lead). **Vindi Jurinovic:** data curation (equal), formal analysis (equal), software (equal), visualization (equal). **Anton Burkhard‐Meier:** writing – review and editing (equal). **Sultan Abdel‐Rahman:** data curation (equal), project administration (equal), writing – review and editing (equal). **Markus Albertsmeier:** writing – review and editing (equal). **Alexander Klein:** writing – review and editing (equal). **Hans Roland Dürr:** writing – review and editing (equal). **Nina‐Sophie Schmidt‐Hegemann:** writing – review and editing (equal). **Thomas Knösel:** writing – review and editing (equal). **Wolfgang G. Kunz:** writing – review and editing (equal). **Emanuel Stutz:** writing – review and editing (equal). **Michael von Bergwelt‐Baildon:** writing – review and editing (equal). **Dorit Di Gioia:** investigation (equal), methodology (equal), writing – review and editing (equal). **Lars H. Lindner:** funding acquisition (equal), investigation (equal), methodology (equal), resources (equal), writing – review and editing (equal).

## Ethics Statement

The Internal Review Board and the Ethical Review Committee at the Ludwig‐Maximilians‐University (LMU) Hospital, Munich, Germany, approved the protocol of this research project (Protocol Nr. 23–0128).

## Consent

Patient consent was waived for this analysis due to its retrospective design and irreversible anonymization of all data. All authors have read and agreed to the published version of the manuscript.

## Conflicts of Interest

The authors declare no conflicts of interest.

## Supporting information


Data S1:

## Data Availability

The data presented in this study are available on specific request from the corresponding author. The data are not publicly available for reasons of data protection and data economy.
