# Supplementary material for: Doxorubicin Plus Dacarbazine Versus Doxorubicin Plus Ifosfamide in Combination With Regional Hyperthermia in Patients With Advanced Leiomyosarcoma: A Propensity Score‐Matched Analysis
Source: Cancer Med. 2025 Feb 25;14(4):e70655. doi: 10.1002/cam4.70655 (PMC11861570; doi:10.1002/cam4.70655)
Supplement: Supplementary file 1 — Data S1: [file CAM4-14-e70655-s001.docx]

|  | **Value** | | **AI** | **AD** | **p-value** |
| --- | --- | --- | --- | --- | --- |
| **Age** |  | Median | 58 | 56 | 0.47 |
| **Sex** | Female | % | 69 | 81 | 0.21 |
|  | Male |  | 31 | 19 |  |
| **Recurrent tumor** | Yes | % | 19 | 9 | 0.16 |
|  | No |  | 81 | 91 |  |
| **Largest diameter** |  | Median | 9 | 8 | 0.19 |
| **Histology** | Uterine LMS | % | 36 | 13 | 0.013 |
|  | Non-uterine LMS |  | 64 | 87 |  |
| **Metastasis at beginning of chemotherapy** | Yes | % | 19 | 17 | 0.83 |
|  | No |  | 81 | 83 |  |
| **Grade** | 3 | % | 51 | 63 | 0.24 |
|  | 2 |  | 49 | 37 |  |
| **Site of primary tumor** | Extremity | % | 22 | 19 | 0.64 |
|  | Intraabdominal |  | 75 | 81 |  |
|  | Trunk |  | 2 | 0 |  |
|  | Head and neck |  | 2 | 0 |  |
| **Site of metastases at beginning of chemoth.** | Liver | % | 18 | 36 | 0.22 |
|  | Pulmonary |  | 64 | 17 |  |
|  | Liver + pulm. |  | 0 | 12 |  |
|  | Other |  | 18 | 34 |  |
| **Surgery** | Yes | % | 97 | 98 | 0.60 |
|  | No |  | 3 | 2 |  |
| **Extent of surgery** | R0 | % | 63 | 48 | 0.025 |
|  | R1 |  | 19 | 46 |  |
|  | R2 |  | 2 | 2 |  |
|  | Rx |  | 16 | 4 |  |
| **Radiotherapy** | Yes | % | 36 | 41 | 0.59 |
|  | No |  | 64 | 59 |  |
| **Radiologic response to neoadjuvant CT** | PR | % | 30 | 7 | 0.10 |
|  | SD |  | 52 | 63 |  |
|  | PD |  | 19 | 30 |  |

**Supplementary File**

Table 1: Baseline characteristics of the matched population (n=101). Displayed are the weighted medians or weighted ratios. The p-value was calculated by the weighted t-test for numerical, and weighted $\chi2$-test for categorical data.


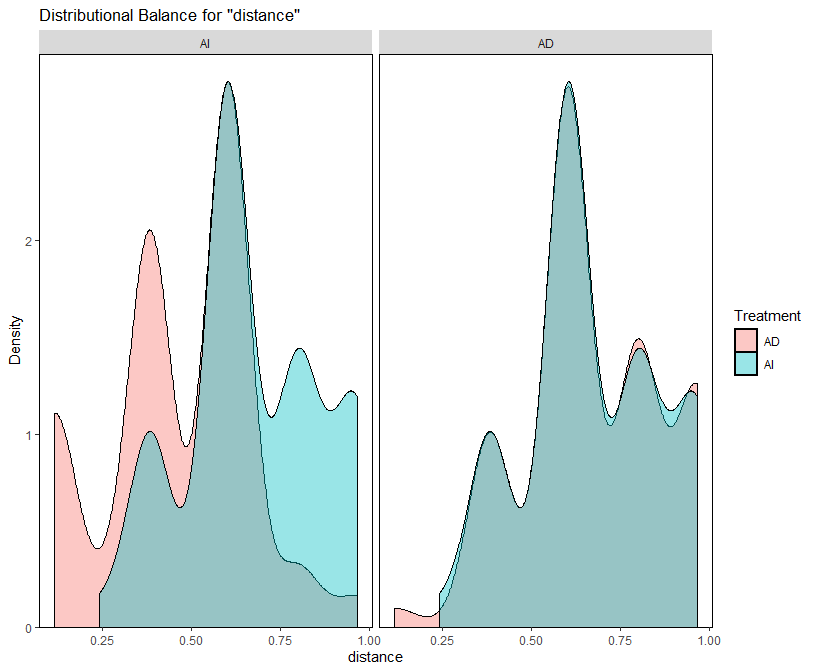


Figure 1: Balance plot / performance of the propensity score matching by using the optimal full matching method.


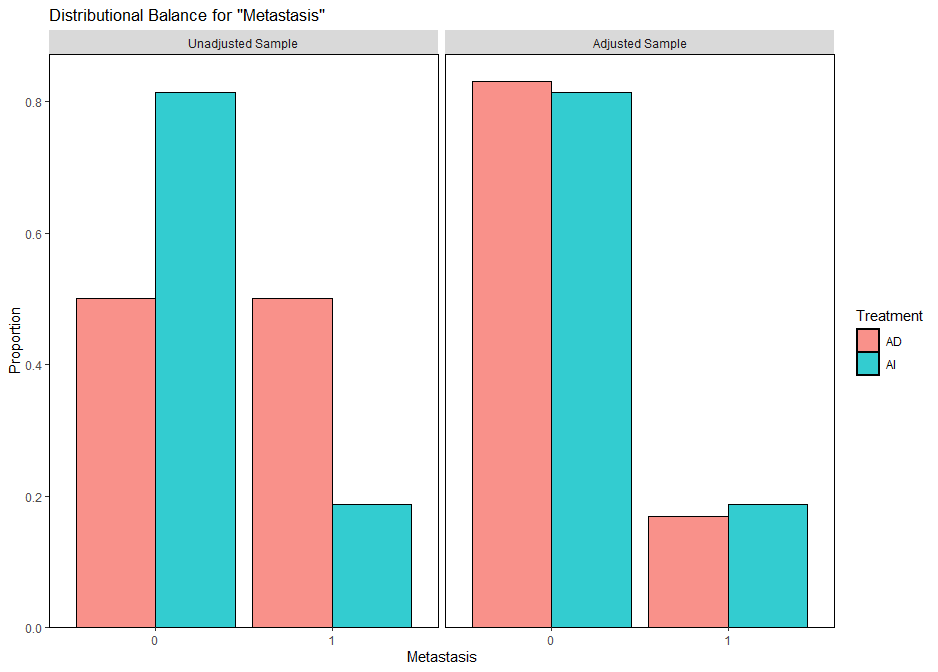


Figure 2: Distribution of primary metastasis before and after propensity score matching.


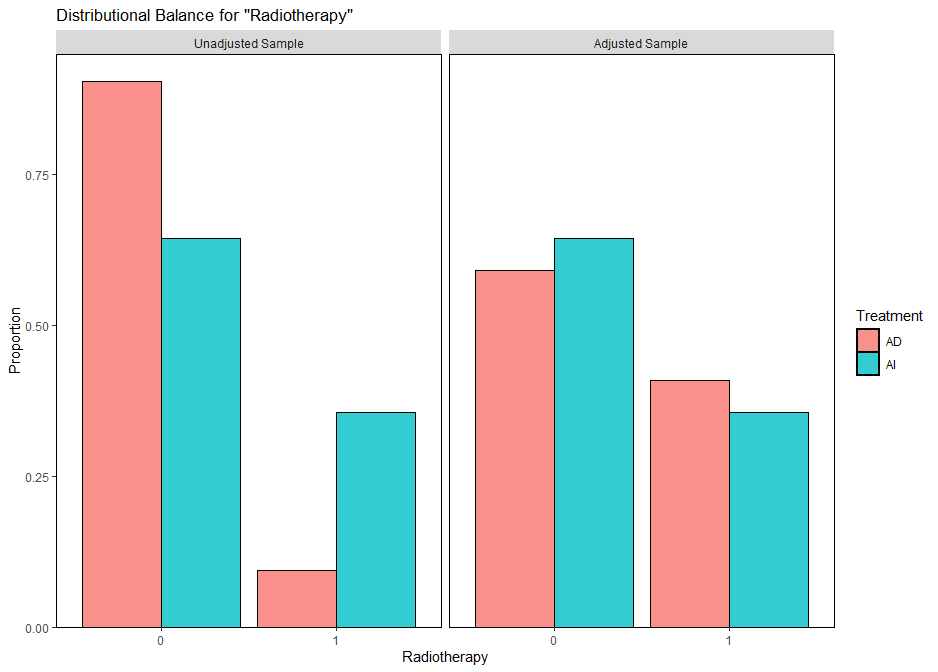


Figure 3: Distribution of radiotherapy before and after propensity score matching.


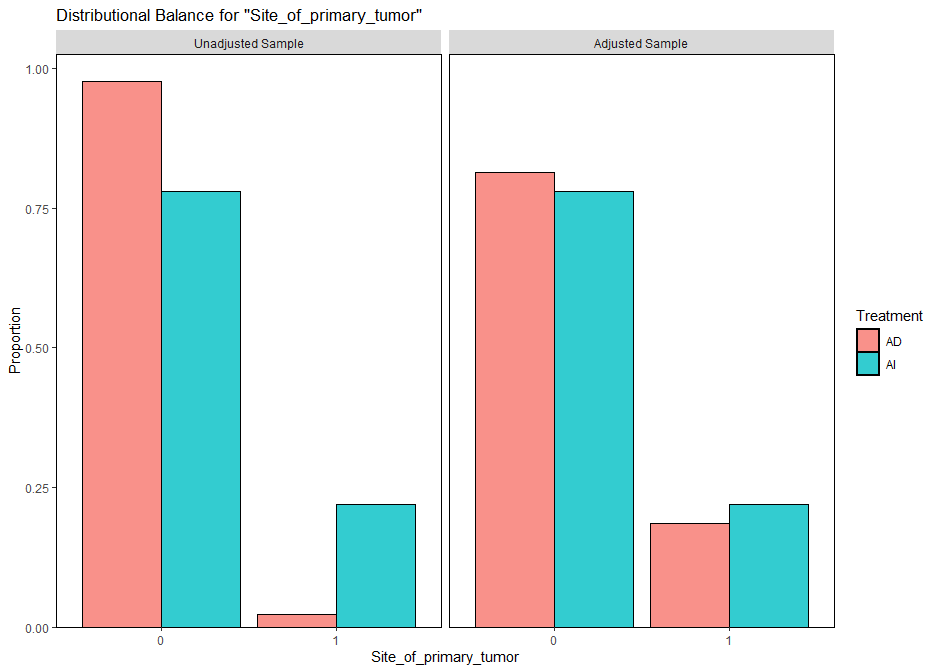


Figure 4: Distribution of primary tumor sites before and after propensity score matching.


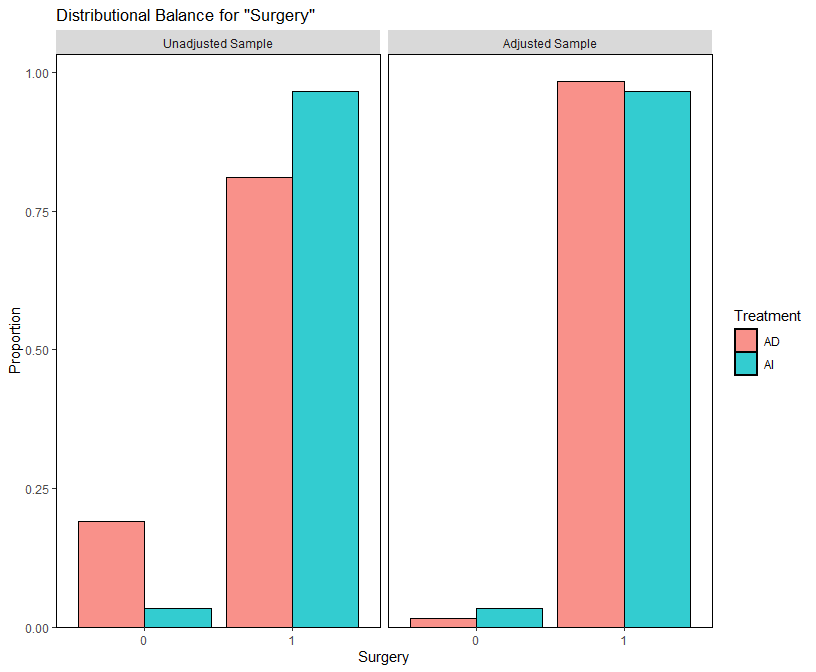


Figure 5: Distribution of surgery before and after propensity score matching.

Table 2: PFS analysis in the exploratory subgroup within the propensity score-matched cohort without presence of metastasis at initial diagnosis.

| **Factor** | **Strata** | **Sig.** | **Hazard Ratio** |
| --- | --- | --- | --- |
| Treatment regimen | AD vs. AI | 0.31 | 0.57 (0.19-1.71) |
| Tumor site | Extremity vs. non-extremity | 0.29 | 0.71 (0.38-1.34) |
| Surgical resection | Yes vs. no | 1.0∙10^-8^ | 0.01 (0.00-0.03) |
| Radiotherapy | Yes vs. no | 0.031 | 0.47 (0.23-0.93) |

Table 3: PFS analysis in the exploratory subgroup within the propensity score-matched cohort with presence of metastasis at initial diagnosis.

| **Factor** | **Strata** | **Sig.** | **Hazard Ratio** |
| --- | --- | --- | --- |
| Treatment regimen | AD vs. AI | 0.11 | 0.55 (0.26-1.15) |
| Tumor site | Extremity vs. non-extremity | 0.0052 | 0.15 (0.04-0.57) |
| Surgical resection | Yes vs. no | 0.14 | 0.61 (0.32-1.17) |
| Radiotherapy | Yes vs. no | 0.039 | 3.77 (1.07-13.26) |

Table 4: OS analysis in the exploratory subgroup within the propensity score-matched cohort without presence of metastasis at initial diagnosis.

| **Factor** | **Strata** | **Sig.** | **Hazard Ratio** |
| --- | --- | --- | --- |
| Treatment regimen | AD vs. AI | 0.37 | 1.77 (0.51-6.12) |
| Tumor site | Extremity vs. non-extremity | 0.0030 | 5.38 (1.77-16.36) |
| Surgical resection | Yes vs. no | NA | NA |
| Radiotherapy | Yes vs. no | 0.098 | 0.37 (0.11-1.20) |

Table 5: OS analysis in the exploratory subgroup within the propensity score-matched cohort with presence of metastasis at initial diagnosis.

| **Factor** | **Strata** | **Sig.** | **Hazard Ratio** |
| --- | --- | --- | --- |
| Treatment regimen | AD vs. AI | 0.64 | 7.88 (0.29-2.13) |
| Tumor site | Extremity vs. non-extremity | 3.7∙10^-124^ | 2.1∙10^-9^ (3.9∙10^-10^ -1.1∙10^-8^) |
| Surgical resection | Yes vs. no | 0.24 | 0.51 (0.16-1.58) |
| Radiotherapy | Yes vs. no | 0.013 | 3.20 (1.28-8.00) |
